# Implementation and evaluation of a quality improvement initiative for central venous catheter management in neonates undergoing therapeutic hypothermia for perinatal asphyxia

**DOI:** 10.1136/bmjpo-2026-004817

**Published:** 2026-09-01

**Authors:** Margreet R Koolen-De Koninck, Tom Ouwehand, Florian Cassel, Sinno HP Simons, Gerbrich E van den Bosch

**Affiliations:** 1 Department of Neonatal and Pediatric Intensive Care, Division of Neonatology, Erasmus MC Universitair Medisch Centrum Rotterdam, Rotterdam, The Netherlands

**Keywords:** Intensive Care Units, Neonatal, Hypoxia, Therapeutics

## Abstract

**Objectives:**

Therapeutic hypothermia (TH) is an effective treatment for hypoxic-ischaemic encephalopathy in infants following perinatal asphyxia, reducing mortality and morbidity when initiated within 6 hours after birth. Both perinatal asphyxia and TH require intensive management and therefore central venous access, such as umbilical venous catheters (UVC) or peripherally inserted central catheters (PICC). Placement of these catheters can lead to a delay in initiating TH. This study evaluated a potential quality improvement using subclavian vein catheters (SVCs) in TH patients, focusing on time to initiation, integration into routine practice and safety.

**Methods:**

We conducted a retrospective study including all neonates treated with TH between July 2017 and April 2025 at a level IV neonatal intensive care unit. Data included catheter type, placement success, adverse events and relation to TH initiation time.

**Results:**

A total of 219 patients were included: 160 prior and 59 after SVC implementation. Between 2017 and 2022, UVC was the most used (50.6%), followed by PICC (21.3%), while 12.5% had unsuccessful catheter placement. Catheter-related difficulties occurred in 51.6% overall and in 46/56 (82.1%) with delayed TH initiation beyond 6 hours. The introduction of the SVC led to an increase in its use up to 69.5% of patients and an overall 100% central catheter success rate. Mean time to initiate TH was 39.4 min shorter when SVC was chosen as the primary catheter (p=0.003), and delayed initiation decreased from 25.6% to 13.6%. No SVC-related adverse events were reported.

**Conclusions:**

Since introducing SVCs, catheter placement success improved and TH initiation was faster, indicating that SVCs are clinically feasible and safe. This quality improvement initiative represents a step forward toward optimising care for neonates undergoing TH. Further prospective studies are needed to confirm these findings and assess long-term outcomes.

**Implications for clinical practice:**

Implementing SVCs as primary central access in neonates undergoing TH can reduce delays and improve care without increasing adverse events.

**WHAT IS ALREADY KNOWN ON THIS TOPIC**

- Starting therapeutic hypothermia within 6 hours after birth is critical for improving outcomes in infants with perinatal asphyxia, yet delays in achieving timely initiation remain common.

**WHAT THIS STUDY ADDS**

- This study shows that challenges with central venous access occur frequently in this patient group. Introducing clinician-placed ultrasound-guided subclavian vein catheters (SVCs) might significantly reduce the time to initiate therapeutic hypothermia and eliminate failed central catheter placements. SVCs were safely integrated into routine neonatal care without procedure-related complications.

**HOW THIS STUDY MIGHT AFFECT RESEARCH, PRACTICE OR POLICY**

- Using SVCs as a primary central access option may improve workflow efficiency, reduce treatment delays and enhance the quality of hypothermia care. These findings support further evaluation of SVCs in clinical guidelines and future research on optimising timely therapeutic hypothermia delivery.

## Introduction

### Problem description

 Perinatal asphyxia may arise before, during or after delivery due to complications such as placental insufficiency or intrapartum events. Resulting oxygen deprivation can impact multiple organ systems, leading to both mortality and major morbidities such as hypoxic ischaemic encephalopathy (HIE), respiratory failure, cardiovascular compromise and acute kidney injury.^1^

Additionally, HIE following perinatal asphyxia can result in unfavourable long-term outcomes such as cerebral palsy, cognitive dysfunction and epilepsy.^2^

Therapeutic hypothermia (TH) reduces mortality and improves neurodevelopmental outcomes when initiated as soon as possible and ideally within 6 hours after birth.^3 4^ However, delays in TH initiation remain common and may result from delayed recognition of HIE, transport times, clinical stabilisation needs or technical and logistical challenges in establishing central venous access.

### Available knowledge

Newborns with perinatal asphyxia undergoing TH require intensive monitoring and circulatory support in a neonatal intensive care unit (NICU) setting. The umbilical venous catheter (UVC), the umbilical arterial catheter (UAC) or peripheral arterial catheter and the peripherally inserted central catheters (PICC) are frequently used to obtain central venous or arterial access in newborn infants suffering from perinatal asphyxia. Both the UVC and UAC are prone to malposition, dislodgement and infection.^5^ Additionally, during insertion, full closure of the cooling suit is not possible, potentially compromising temperature management. PICCs are another common option, but their placement can be technically challenging and time-consuming after the initiation of TH due to hypothermia-induced vasoconstriction.^6^

Exploring alternative options may reduce some of the challenges faced in central venous access in neonatal TH patients. Subclavian venous catheters (SVCs) could potentially offer more rapid, direct central access, be less affected by vasoconstriction and allow improved use of the cooling suit during placement, making them a potentially attractive option for timely and reliable access in TH-patients. However, SVC placement requires training, specialised echo equipment and is not without risks. Potential disadvantages include mechanical complications such as pneumothorax, arterial puncture, cardiac tamponade and malposition.^7^

#### Rationale

Persistent catheter-related delays in TH initiation were observed in our NICU. The introduction of SVCs was proposed to improve reliability of central access, reduce delays and streamline care within existing workflows. Based on emerging evidence of high procedural success and low complication rates in neonates,^8^ we hypothesised that SVCs could be safely integrated into practice.

#### Specific aim

The aim of this quality improvement initiative was to evaluate the clinical feasibility, integration into routine practice, safety and potential impact of SVC implementation on the timeliness of therapeutic hypothermia initiation in neonates with perinatal asphyxia.

## Methods

### Context

The introduction of ultrasound-guided SVC placement performed by neonatologists was implemented at the level IV NICU of Erasmus MC-Sophia, Rotterdam. This unit serves a large referral region with long and variable transport distances. The Dutch perinatal system features decentralised delivery units and limited capacity for active cooling during transport. These structural factors contribute to variability in pre-NICU timelines and highlight the need for in-hospital improvements to reduce delays.

### Intervention

As of January 2023, three neonatologists were trained to insert ultrasound-guided SVCs in the clinical setting. Ultrasound-guided subclavian vein catheterisation was performed under sterile conditions by a trained neonatologist. After identification of the subclavian vein using real-time ultrasound, the vein was punctured under sterile conditions, followed by insertion of a guidewire and catheter advancement using the Seldinger technique. Catheter type and size were selected based on patient characteristics.

The primary choice of central catheter type from that point on was determined by the attending neonatologist at admission to the NICU, based on the availability of a trained physician. The catheters used included the Vygon Nutriline 2Fr/3Fr/4Fr single-lumen and double-lumen polyurethane (PUR) catheters, Premicath 1Fr, Twinflow 2Fr and Multicath 2 3Fr. All catheter tip positions were confirmed using radiographic imaging following placement. The intervention did not replace UVC or PICC use but expanded the repertoire of viable central access methods, prioritising SVCs when feasible. Therapeutic hypothermia was initiated using the Criticool full-body cooling system.

### Study of the intervention

We conducted a retrospective cohort study including all neonates treated with TH between July 2017 and April 2025. The pre-implementation period (July 2017 to December 2022) relied primarily on UVC/PICC access; the post-implementation period (January 2023 to April 2025) included the SVC option.

### Measures

Primary outcome:

- Time between onset of asphyxia and initiation of active TH (minutes) as registered in the electronic patient record in relation to the primary choice of central venous catheter, from the implementation of the SVC in January 2023 up until April 2025.

Secondary outcomes:

- Success rate of central catheter placement by type.
- Proportion of infants meeting the ≤6-hour TH initiation target.
- SVC-related adverse events (arterial puncture, pneumothorax, thrombosis, infection).

All data were extracted from the electronic medical record (ChipSoft HiX) via automated query and manual validation.

### Analysis

Continuous variables were summarised as mean±SD or median (IQR). Categorical variables were described as counts and percentages. Comparisons used Student’s t-tests for continuous variables and χ^2^ tests for categorical variables.

Linear regression assessed the association between primary catheter type (SVC vs UVC), TH initiation time and time from birth to NICU admission.

Regression assumptions were verified through inspection of histograms and normal P–P plots of standardised residuals (normality); residuals-versus-fitted plots (homoscedasticity) and partial regression plots (linearity). All analyses were performed using SPSS (V.28.0.1.0 (142)). This study was reported in accordance with the Standards for Quality Improvement Reporting Excellence (SQUIRE) 2.0 guidelines for quality improvement reporting.^9^

### Patient and public involvement

Patients or members of the public were not involved in the design, conduct, reporting or dissemination plans of this study. This retrospective analysis used routinely collected clinical data, and no direct patient involvement was feasible or required.

## Results

### Patient characteristics and setting

**Figure 1:**
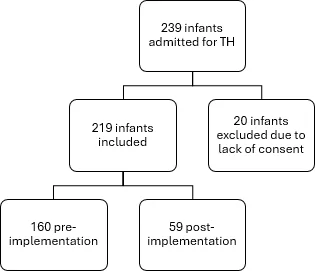
Inclusion flowchart/flowchart illustrating patient inclusion for the study. Of 239 infants admitted for TH, 20 were excluded due to lack of consent, resulting in 219 included infants. These were divided into two groups: 160 pre-implementation and 59 post-implementation of subclavian vein catheters. TH, therapeutic hypothermia.

Of the 239 infants admitted for TH, 219 (92%) were included in this study, 160 prior to the implementation of SVC and 59 afterwards (figure 1). 129 (59%) of those patients were male, with a mean birth weight of 3286±593 g and a median gestational age of 39.6 weeks (IQR 3.0). The majority were born outside our hospital (n=201; 91%); by caesarean section (n=197; 51%) and had a median Apgar score at 5 min of 3 (IQR 4.0) (table 1). Time between birth and admission to the Erasmus MC NICU ranged widely from 0 to 549 min (mean 195.7±92.4 min).

**Table 1:** Patient characteristics

	Pre-implementation	Post-implementation	P
	(N=160)	(N=59)	
Birth weight (g)			
Mean	3302±547	3245±708	0.53
Sex			
Male	98 (61.3%)	31 (52.5%)	0.25
Gestational age (weeks)			
Median (Min, Max)	39 2/7 (34 1/7, 42 1/7)	40 0/7 (34 6/7, 42 0/7)	0.17
Apgar score <3 at 5 min			
<3 (%)	82 (52.9)	31 (57.4)	0.567
Respiratory support at admission			
None	13 (8.1%)	2 (3.4%)	0.323
Non-invasive ventilation	45 (28.2%)	14 (23.8%)	
Invasive ventilation	102 (63.7%)	43 (72.9%)	
C-section			
Yes	75 (46.9%)	32 (54.2%)	
Place of birth			
Home birth	13 (8.1%)	10 (16.9%)	0.06
Level IV NICU	16 (10.0%)	2 (3.4%)	
Level I/II hospital	131 (81.9%)	47 (79.7%)	
Location before transfer to NICU			
Home	11 (6.9%)	5 (8.5%)	0.22
Level IV NICU	16 (10%)	2 (3.4%)	
Level I/II hospital	133 (83.1%)	52 (88.1%)	
Time between birth and NICU admission (minutes)			
Mean	191.21±95.13	207.80±84.07	0.24
Deceased			
Yes (%)	38 (23.8)	10 (16.9)	

NICU, neonatal intensive care unit.

### Initiation of therapeutic hypothermia

**Figure 2:**
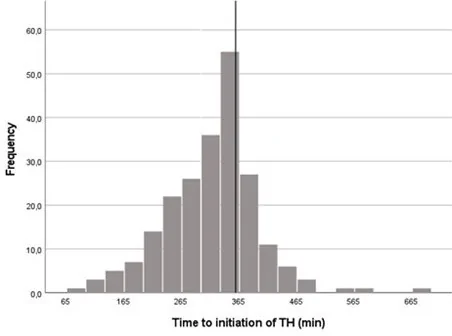
Histogram showing the distribution of time to initiation of therapeutic hypothermia (TH) in minutes for all patients. The data are centered around approximately 365 minutes, with most cases falling between 265 and 465 minutes.

Across the full cohort of 219 patients, time to initiation of TH ranged from 90 to 670 min, with a mean of 319.5±80 min (figure 2). Delayed initiation, defined as exceeding the 6-hour target, occurred in 56 cases (25.6%), with a median delay time of 24 min (IQR 58.5). The most frequently reported reasons for delay were major catheter placement issues (n=40; 73.7%), time between birth and NICU admission exceeding 6 hours (n=9; 16.1%), clinical instability (n=3; 5.4%), missing equipment (n=2; 3.6%) and prolonged assessment of neurological condition (n=2; 3.6%).

### Primary outcome: time to initiation of TH

**Figure 3:**
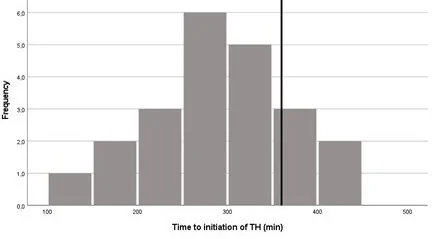
Histogram showing the distribution of time to initiation of therapeutic hypothermia (TH) in infants with primary subclavian vein catheter (SVC) placement. Most cases cluster between approximately 250 and 400 minutes, with a peak around 300 minutes.

**Figure 4:**
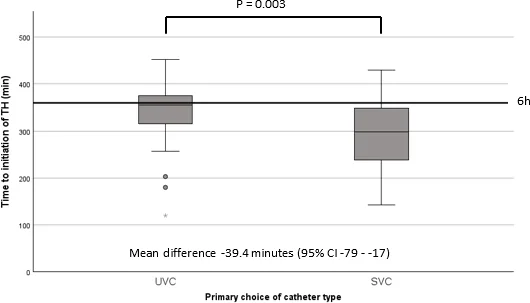
Boxplot of time to initiation of TH by primary central venous catheter/boxplot comparing time to initiation of TH between infants with primary UVC and those with primary SVC placement. Median initiation time was 298 min for the SVC group versus 356 min for the UVC group, with a mean difference of 39.4 min (95% CI −79 to −17; p=0.003). SVC, subclavian venous catheters; TH, therapeutic hypothermia; UVC, umbilical venous catheter.

A linear regression examined the association between primary catheter choice (SVC vs UVC) and time to initiation of TH, adjusting for time between birth and admission. The model was statistically significant, *F*(3,55)=12.548, p<0.001; adjusted R²=0.374. Both catheter choice (p=0.003) and birth-to-admission time (p<0.001) significantly affected time to TH initiation. The mean time to TH decreased by 39.4 min when an SVC was primarily inserted (95% CI −79.1 to −16.5) (table 2, figures 3 and 4). Delayed onset beyond 6 hours decreased to 13.6% in primary SVC-patients, with a 2.8-fold increase in odds of timely TH (Pearson χ^2^, p=0.03) (table 3).

**Table 2:** Linear regression of time to initiation of TH—primary choice of SVC

	Unstandardised coefficients		Standardised coefficients	t	Significance	95.0% CI	
	B	SE	Beta			Lower bound	Upper bound
(Constant)	240.467	20.594		11.677	<0.001	199.212	281.722
Primary SVC	−47.775	15.634	−0.32	−3.056	0.003	−79.094	−16.456
Time between birth and NICU admission	0.492	0.091	0.569	5.425	0.001	0.31	0.674

Dependent variable: time to initiation of TH (min).

NICU, neonatal intensive care unit; SVC, subclavian venous catheters; TH, therapeutic hypothermia.

**Table 3:** Pearson χ^2^ test; SVC as primary catheter and >6 hours postpartum initiation of TH

Test statistics (χ²)	df	P value	OR	95% CI
4.71	1	0.03*	2.78	0.94 to 8.2

SVC, subclavian venous catheters; TH, therapeutic hypothermia.

### Secondary outcomes

From 2017 to 2022, the UVC was the standard first-choice central catheter. It was attempted in 152 infants (95%) and successfully inserted in 81 (51%). Among the remaining 49% with an unsuccessful initial attempt, 34 infants (21%) received a PICC, while 25 (16%) required a jugular, subclavian or femoral central catheter placed by the attending anaesthetist. Catheter-related difficulties-including mispositioning and failed attempts-occurred in 84 of 160 patients (52.5%) overall, and in 31 of 38 infants (81.6%) who experienced delayed TH initiation beyond 6 hours. Completely unsuccessful catheter placement occurred in 20 patients (12.5%).

**Figure 5:**
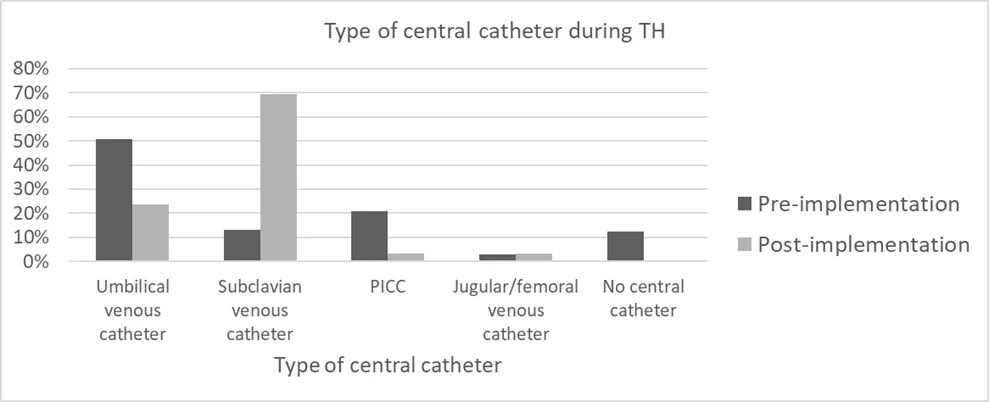
Type of central catheter during TH/bar chart showing the distribution of central catheter types used during TH before and after SVC implementation. PICC, peripherally inserted central catheters; SVC, subclavian venous catheters; TH, therapeutic hypothermia.

After introduction of the SVC, choice for UVC as primary catheter decreased to 63% (n=37), with successful placement in 24% (n=14). The SVC surpassed the UVC as primary choice (n=22, 37%). After failed UVC placement, 19 patients (32%) received an SVC as secondary option, while 2 patients (3%) received a PICC and 2 patients (3%) a femoral or jugular venous catheter. Overall central catheter success increased to 100% (figure 5).

### Unintended consequences

No serious adverse events related to SVC placement-such as arterial or pleural puncture-were reported. One asymptomatic jugular venous thrombosis was incidentally detected on MRI, without clinical sequelae. No catheter-related infections (positive blood cultures within 1 week) were reported. No unexpected workflow disruptions or other unintended consequences occurred following SVC implementation.

### Missing data

No missing data were reported for the primary outcome, catheter type, complications or reasons for delay. All included patients had complete data for the relevant variables.

## Discussion

### Key findings

This retrospective cohort study evaluated the clinical feasibility of placement by neonatologist of SVCs in neonates undergoing TH, with a particular focus on their potential impact on the time to initiation of therapy. Our findings demonstrate that SVC placement was successfully integrated into routine care without adverse events and was significantly associated with a reduction in the time to initiation of TH. Although the absolute reduction in time was only 39 min, this represents a clinically meaningful improvement within the strict 6-hour therapeutic window for TH initiation, where even modest delays may impact neuroprotective efficacy. These results support the relevance of catheter choice within the TH workflow, as delays in starting TH were common, and catheter-related difficulties were a major contributor.

To our knowledge, this is the first study to explore both the potential delay in initiating TH and the specific role of central catheter type in this context. The data provide insight into the frequency and causes of such delays and suggest that optimising central venous access may be a practical approach to improving the timeliness of TH. Although evidence regarding the benefits of ultra-early cooling remains mixed, recent studies indicate potential improvements in MRI outcomes and seizure burden when TH is started as early as possible.^4 10^ This reinforces the clinical imperative to minimise delays in initiating therapy. Compared with the recent work by Mistry *et al*,^11^ who reported delays in 54% of infants and achieved reductions through active cooling during transport-our delay rate was lower, yet still notable. Their study did not describe the use of central venous catheters or the influence of access type on workflow, which limits direct comparison of procedural approaches.

Active cooling during transport has not been implemented in our region due to logistical and organisational constraints. The Dutch perinatal care system is characterised by a relatively high proportion of home births, a decentralised structure with many level I and II units and highly variable transfer distances to tertiary NICUs. These contextual factors make it challenging to standardise early cooling practices across referring centres. Within this complex care landscape, choosing primary SVC access may represent a modest but meaningful step toward reducing procedural delays and supporting more streamlined care. In addition, simultaneous initiation of hypothermia during vascular access appeared more feasible during SVC placement than during UVC placement, where optimal use of the cooling blanket and maintenance of sterile conditions may be more challenging.

Our findings regarding the feasibility and safety of SVCs are consistent with previous reports, such as the study by Lausten-Thomsen *et al*,^8^ which also found high success rates without adverse events. In our cohort, SVC placement was uniformly successful and free of major complications, reinforcing its suitability within the neonatal TH pathway. A strength of the present study is that it describes a potential system-level improvement based on an intervention that is relatively simple to implement and requires only limited structural changes once staff are trained.

Nevertheless, several limitations must be considered. The retrospective design relies on clinical documentation recorded during acute care, which may introduce inaccuracies in timing or procedural details. Time to initiation of TH was the only feasible measure, as continuous temperature recordings were not available, preventing analysis of time to reach target temperature. Furthermore, we were unable to assess the specific procedural times for UVC and SVC placement, which may provide further insight into workflow efficiency and the relative contribution of each technique to overall delay. The decision to place an SVC depended on the availability of a neonatologist trained in the procedure, introducing an element of randomness that may have influenced catheter choice and internal validity. Only three neonatologists were trained in SVC placement, which may have limited consistency and the generalisability of results. This reliance on a limited number of trained operators may also affect scalability; however, training efforts within our unit are ongoing to expand proficiency and improve sustainability. Increased awareness among staff during the study period may also have influenced clinical behaviour, potentially contributing to improved outcomes independently of the intervention.

Despite these limitations, the findings suggest that SVCs can be safely integrated into neonatal TH practice and may help reduce delays in initiating treatment. The intervention appears sustainable within our setting, and the approach may be transferable to other contexts where logistical constraints limit the feasibility of early or transport-based cooling. This exploratory work provides a foundation for further improvement in the TH pathway. Future studies should prospectively evaluate the association between catheter type and time to TH initiation, including procedural details such as SVC placement duration and workflow sequences and consider how SVC-based strategies could be combined with other early-cooling interventions across different healthcare systems.

## Conclusion

The use of SVCs was successfully introduced without adverse events and showed its potential for quality improvement by significantly reducing time to initiate therapeutic hypothermia. Future prospective studies are needed to confirm our findings and to study the potential improved outcome of infants by using SVCs.

## Data Availability

Data are available upon reasonable request.
