# Supplementary material for: Implementation and evaluation of a quality improvement initiative for central venous catheter management in neonates undergoing therapeutic hypothermia for perinatal asphyxia
Source: BMJ Paediatr Open. 2026 Sep 1;10(1):e004817. doi: 10.1136/bmjpo-2026-004817 (PMC13536075; doi:10.1136/bmjpo-2026-004817)
Supplement: Supplementary file 1 [file bmjpo-2026-004817.reviewer_comments.pdf]

PEER REVIEW HISTORY

BMJ Paediatrics Open publishes all reviews undertaken for accepted manuscripts. Reviewers are asked to complete a checklist review form and are provided with free text boxes to elaborate on their assessment. These free text comments are reproduced below.

ARTICLE DETAILS

**TITLE (PROVISIONAL)**	"Implementation and evaluation of a quality improvement initiative for central venous catheter management in neonates undergoing therapeutic hypothermia for perinatal asphyxia"
**AUTHORS**	Koolen-De Koninck, Margreet R.<br>Ouwehand, Tom<br>Cassel, Florian<br>Simons, Sinno<br>van den bosch, Gerbrich

VERSION 1 – REVIEW

**REVIEWER NAME**	Kasala, Sweatha
**REVIEWER AFFILIATION**	Baylor Scott and White Central Texas
**REVIEWER CONFLICT OF INTEREST**	No Conflict of Interest
**AI DISCLOSURE**	No, I have not used AI
**DATE REVIEW RETURNED**	21-Apr-2026

**GENERAL COMMENTS**	It would be interesting to track the time to placement of a UVC to the SVC .

**REVIEWER NAME**	Avvaru, Parthu
**REVIEWER AFFILIATION**	Akron Children's Hospital, Neonatal and Perinatal Medicine
**REVIEWER CONFLICT OF INTEREST**	No Conflict of Interest
**AI DISCLOSURE**	No, I have not used AI
**DATE REVIEW RETURNED**	21-Apr-2026

**GENERAL COMMENTS**	I appreciate the team for mastering SVC placement and achieving time savings for initiating hypothermia. Over all, great study, certainly helps to add it to the literature as SVC might be a reasonable alternative for achieving central access in neonates.<br>My questions: Is SVC placement ultrasound guided? What is the average time needed to place a SVC? What equipment is used to initiate hypothermia? With some cooling blanket technology, umbilical venous lines can be placed simultaneously while achieving hypothermia.

VERSION 1 – AUTHOR RESPONSE

REVIEWER 1 COMMENTS:
It would be interesting to track the time to placement of a UVC to the SVC .
We thank the reviewer for the careful reading of our manuscript and for this insightful suggestion.
Due to the retrospective design of our study, detailed procedural timing was not consistently recorded

and therefore could not be reliably analysed. We agree that this represents a clinically relevant metric, as it would provide further insight into workflow delays and decision-making during vascular access. We have acknowledged this as a limitation in the Discussion section on page 8 and highlighted it as an important area for future prospective evaluation.

REVIEWER 2 COMMENTS:
I appreciate the team for mastering SVC placement and achieving time savings for initiating hypothermia. Over all, great study, certainly helps to add it to the literature as SVC might be a reasonable alternative for achieving central access in neonates.
We thank the reviewer for the positive and encouraging feedback on our manuscript and for these thoughtful and relevant questions. Please find our detailed answers below.
My questions:
1. Is SVC placement ultrasound guided?
Yes, SVC placement in our unit is performed using ultrasound guidance, with subsequent radiographic confirmation of catheter tip position. This has now been clarified in the Methods section on page 4. It now reads as follows: “Ultrasound-guided subclavian vein catheterization was performed under sterile conditions by a trained neonatologist. After identification of the subclavian vein using real-time ultrasound, the vein was punctured under sterile conditions, followed by insertion of a guidewire and catheter advancement using the Seldinger technique. Catheter type and size were selected based on patient characteristics.” “All catheter tip positions were confirmed using radiographic imaging following placement. “

2. What is the average time needed to place a SVC?
Due to the retrospective nature of this study, detailed procedural timing data for SVC placement were not consistently recorded and could therefore not be reliably analysed. We agree that this is a relevant parameter and have acknowledged this limitation in the Discussion, highlighting the need for prospective evaluation of procedural timing.

3. What equipment is used to initiate hypothermia? With some cooling blanket technology, umbilical venous lines can be placed simultaneously while achieving hypothermia.
In our unit, therapeutic hypothermia is initiated using the Criticool® full-body cooling system. This has now been clarified in the Methods section on page 4.
We agree that with this cooling technology, some vascular access procedures such as SVC placement may be performed simultaneously with the initiation of TH. In our experience, this was more readily feasible when SVC placement was chosen, and this may have contributed to the observed reduction in time to initiation of therapy. This reflects the relative ease with which SVC placement can be integrated into ongoing care while maintaining optimal use of the cooling system and procedural conditions. In contrast, during UVC placement the cooling blanket or wrap cannot be used optimally, as it needs to be at least partially opened to allow adequate access to the umbilical area. This makes it more difficult to maintain a sterile field, and the procedural complexity and positioning requirements of UVC placement may further limit the feasibility of simultaneous workflows.
We have added a statement in the Discussion on page 7 to highlight that simultaneous initiation of hypothermia appears more feasible during SVC placement than during UVC placement, which may partly explain the observed improvement in time to initiation of therapy. It now reads: “In addition, simultaneous initiation of hypothermia during vascular access appeared more feasible during SVC placement than during UVC placement, where optimal use of the cooling blanket and maintenance of sterile conditions may be more challenging.”

VERSION 2 – REVIEW

**REVIEWER NAME**	Kasala, Sweatha
**REVIEWER AFFILIATION**	Baylor Scott and White Central Texas
**REVIEWER CONFLICT OF INTEREST**	No Conflict of Interest
**AI DISCLOSURE**	No, I have not used AI
**DATE REVIEW RETURNED**	15-Jul-2026

**GENERAL COMMENTS**	The study addresses a genuinely useful question—whether clinician-placed, ultrasound-guided subclavian vein catheters (SVCs) can reduce delays in initiating therapeutic hypothermia (TH)—and reports a plausible, safety-conscious quality improvement (QI) initiative.

**REVIEWER NAME**	Avvaru, Parthu
**REVIEWER AFFILIATION**	Akron Children's Hospital, Neonatal and Perinatal Medicine
**REVIEWER CONFLICT OF INTEREST**	No Conflict of Interest
**AI DISCLOSURE**	No, I have not used AI
**DATE REVIEW RETURNED**	19-Jul-2026

**GENERAL COMMENTS**	all of my questions were addressed
